# A strategy to digitise natural history collections with limited resources

**DOI:** 10.3897/BDJ.8.e55959

**Published:** 2020-10-23

**Authors:** Joaquim Santos, Paulo Rupino da Cunha, Fátima Sales

**Affiliations:** 1 University of Coimbra, Centre for Functional Ecology - Science for People & the Planet, Department of Life Sciences, Coimbra, Portugal University of Coimbra, Centre for Functional Ecology - Science for People & the Planet, Department of Life Sciences Coimbra Portugal; 2 University of Coimbra, Centre for Informatics and Systems of the University of Coimbra, Department of Informatics Engineering, Coimbra, Portugal University of Coimbra, Centre for Informatics and Systems of the University of Coimbra, Department of Informatics Engineering Coimbra Portugal

**Keywords:** accelerating herbarium digitisation, automate databasing processes, crowdsourcing platform

## Abstract

The present work is a contribution towards accelerating the digitisation process of natural history collections, usually a slow process. A two-stage process was developed at the herbarium of the University of Coimbra: (i) a new workflow was established to automatically create records in the herbarium master database with minimum information, while capturing digital images; (ii) these records are then used to populate a web-based crowdsourcing platform where citizens are involved in the transcription of specimen labels from the digital images. This approach simplifies and accelerates databasing, reduces specimen manipulation and promotes the involvement of citizens in the scientific goals of the herbarium. The novel features of this process are: (i) the validation method of the crowdsourcing contribution that ensures quality control, enabling the data to integrate the master database directly and (ii) the field-by-field integration in the master database enables immediate corrections to any record in the catalogue.

## Introduction

Biological collections are major sources of valuable information with potential for multiple areas of knowledge (Funk 2018). An exponential growth has been noted of publications on subjects as diverse as taxonomy, global change biology or DNA analyses, to name but a few, based on the collections housed in herbaria (Heberling et al. 2019). Such an increase is possible because of the mass digitisation that herbaria have gone through over the last two decades, facilitating the access to specimens and associated information in online catalogues and also in data aggregators, such as GBIF (Soltis 2017). There are several approaches to establish a digitisation plan, depending on the collection characteristics, such as its size, the available resources and budget (Vollmar et al. 2010, Walton et al. 2020). In general, digitisation is a demanding task, requiring considerable human labour and time. Primarily, it consists of creating a record in a database for each specimen and then transcribing the relevant data to specific fields (databasing). A unique identifier is assigned to each specimen in the database and, in most collections, a barcode sticker is placed on the specimen's mounting support. For many collections, it is desirable to capture images of the specimens (imaging). Institutions have established digitisation workflows according to their requirements and preferences (Tulig et al. 2012, Haston et al. 2012, Nieva de la Hidalga et al. 2020).

The herbarium of the University of Coimbra (COI, http://www.uc.pt/en/herbario_digital) is digitising its plant collection of ca. 800,000 specimens and making the data available online (http://coicatalogue.uc.pt). Due to the slowness of the methods only ca. 10% of the materials is processed so far. Some institutes have implemented crowdsourcing agendas to benefit from remote transcribing by volunteers, this proving to be a practical solution (Swanson et al. 2016) with collateral benefits, such as the improved scientific literacy of the public involved (Cronje et al. 2011, Ellwood et al. 2015). Various crowdsourcing platforms have been designed to build on citizen collaboration, from broad scope orientated projects (Zooniverse 2020), to narrow fields of interest, such as biological collections (DigiVol 2020, DoeDat 2020) and even to specific kinds of collections (Les herbonautes 2020). The workflow of all those platforms is similar:

1. creation of a project/mission limited to a number of objects and/or time,
2. submission of data,
3. validation and
4. integration of the collected data in the collection database. The integration of data occurs only when the project is completed.

The software used at COI for databasing is SPECIFY (Specify Collections Consortium 2019), a full suite to manage biological collections, currently used by ca. 500 institutes in more than 40 countries (Specify Collections Consortium 2017). Imaging is made using either an inverted A3 scanner or a full-frame digital photography set-up, depending on the purpose. The filename given to the image file is the specimen's barcode, which enables the automatic association of the image to the corresponding specimen. Images can be acquired in different file formats; therefore, it is necessary to process them when uniformity is required. Image processing is associated with backup workflows; therefore, there is a routine that deals with images to complete all tasks needed.

To speed up digitisation, COI has established a new two-stage workflow that optimises the time spent in the imaging process and associated handling of the specimens. When imaging, the corresponding record in the database is automatically created with minimal information (barcode and taxon). This image is provided to remote volunteers who will transcribe the data from the specimen labels (visible in the image) to corresponding fields in a web form using a dedicated crowdsourcing platform. This platform was developed from scratch to fulfil specific requirements, the main one being the possibility of data integration with the master database in near real time.

## Methodology

**Stage 1. Automatic process to create records with minimum information in the master database from sets of digital images**

The objectives to accomplish during the first stage of the accelerating process are:

- to create records in the master database and
- to process images for file format transformation and backup.

Specimens are stored in the herbarium cabinets according to a taxonomic sequence; therefore, all those in the same folder belong to the same taxon (scientific name). When imaging the specimens, the operator can easily create directories named after the corresponding taxon and store the images produced in the imaging station computer. Each specimen sheet will have one image file, named using a barcode scanner to read all the barcodes on the specimen sheet (there can be more than one specimen per sheet). At the end of an imaging session, each directory will contain one to several image files, each file being named according to the corresponding sheet barcodes. When images are processed to integrate the centralised file system for image storage, a script can be executed to create a record in the master database for each specimen, assigning one determination with the respective taxon name (directory name).

The concept is simple, but some aspects must be considered, depending on the local set-up. The database schema of SPECIFY has more than one hundred tables, so creating records externally to its own interface should be well planned to avoid data corruption.

**Figure 1.**
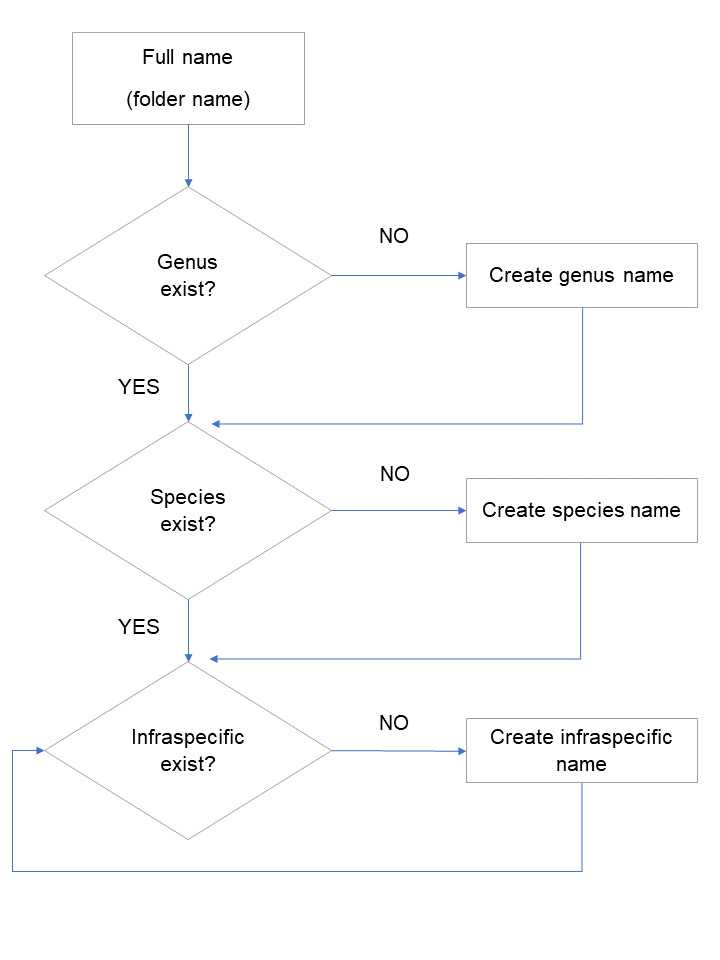
Create taxon name process. This process is called by the main process (Fig. 2) if the full taxon name is not found. A taxon name can have several infraspecific ranks, which are processed using the same routine in the final loop.

The database structure requires the use of a hierarchically-structured taxon tree (Family -> Genus -> Species -> …). Creating determinations from a string being converted to a hierarchical structure must consider all the ranks, including those below species level, i.e. subspecies, variety, form and even other less frequently as microgene. According to botanical nomenclature, an infraspecific taxon includes a connecting term in the full name to denote the rank (subsp. var. etc.) (Turland et al. 2018). The operator will write the name as it is on the specimen folder (author names are not included, as this would increase substantially the complexity for infraspecific names recognition and there will hardly exist homonyms in our collection). Therefore, when the string containing the taxon name is read, the infraspecific rank is standardised to meet what is established in our database, for example, “ssp.” will be converted to “subsp.”. Then, a query is executed in the SPECIFY database to check whether the full name already exists. If a match is not found, the string is split by spaces and the possible name combinations are searched and matched until all the names exist in the hierarchy tree: check and match the genus or create; check and match the species or create; check and match each infraspecific rank or create (Fig. 1).

Thus, when a name does not exist in a rank, it must be created. A given taxon name starts with the generic name. Consequently, all infra-generic names can be created as long as the generic name is in the database. To enable the creation of a new generic name without knowing its family, a new “family” was added in SPECIFY to include all such genera. Later, these can be easily allocated to the right family using SPECIFY tools – with no loss of the determinations created under those genera.

**Figure 2.**
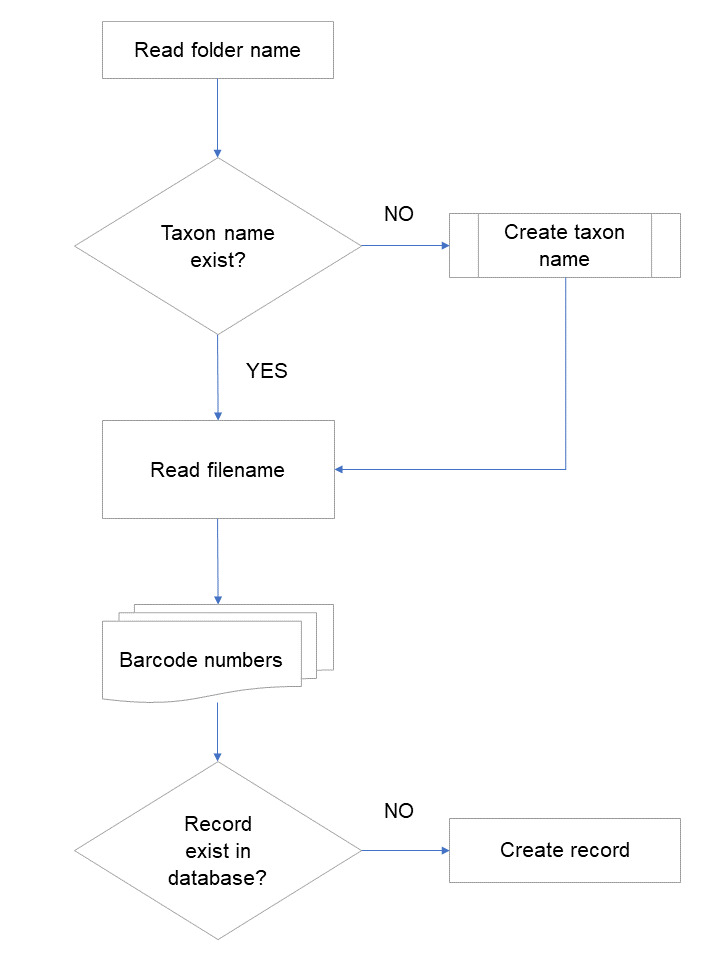
Process to create records in the database from specimen images. If there is the need to create a taxon in the database, the "create taxon name" process is called (Fig. 1). One specimen can have multiple barcodes, which are transcribed in the image filename. Each barcode will correspond to one record in the database, with one determination.

When the taxon name exists in the database, a new record is created for each barcode read from the image filenames inside this one folder. A particular instance is when more than one specimen is mounted on the same sheet. Since the beginning of our digitisation programme, the policy for these cases has been to capture and store only one image of the herbarium sheet. The filename given to this image file contains all the barcodes on the sheet (for example, COI00057276COI00057277.jpg). To cope with this, a routine will split the filenames to obtain all the barcodes in each sheet and create the corresponding record in the database (Fig. 2).

Over time, a specimen can be imaged more than once and all versions are kept because the most recent one is not necessarily that with the greatest detail. For image processing, the first step verifies whether images with the same name already exist in the final destination (not considering file extension). In this case, a suffix is added to the filename (e.g. _1, _2) to avoid overwriting previously-captured images. Then, the original file will be saved to the destination. File format conversions occur if needed and copies will then be created (JPGs, thumbnails).

The PHP language was chosen to perform these operations because it is independent of the operating system and includes functions to copy files, manage images and create records in the database. In addition, the script can be deployed from client machines using only an internet browser. To process Tiff images, Irfanview (Skiljan 2018), a third-party software with command line options, is used, embedded in the PHP code.

**Stage 2. Crowdsourcing platform for specimen label transcription**

This platform was designed to enable citizens-users to transcribe the information on specimen labels into a web form (https://coicatalogue.uc.pt/explorator). The tools already developed with this purpose (e.g. Les herbonautes, DigiVol, Zooniverse) lack some features that we consider essential. The most relevant are:

**To allow users to insert and edit data on either one field or all fields at a time.** Filling in all specimen fields is time-consuming and can be a tedious job. It is also potentially a difficult task, requiring a broad knowledge of taxon names, geographical names, habitat and descriptive terms. Providing one field at a time is a good strategy to maintain users’ attention. Once a field is submitted, the next one is displayed. On each submission, all values are stored in the database to allow the user to skip or leave the task at any moment. Nevertheless, an advanced user may prefer to submit all required fields in a single operation and a tab selector provides this opportunity.

**To compare inputs for the same record from different users and to issue alerts for mismatches.** When users submit a form, the value for each field is compared to previously-submitted values. If a discrepancy is detected, an alert is shown indicating all submissions. The user has the option to return to the field to submit a new answer or to continue to the next field, keeping the initial value. This immediate correction has the obvious purpose of accelerating the validation, but it also has the purpose of educating the users and accelerating their learning process.

**To rank users based on proficiency (categories).** Registered users are assigned a category based on their experience, which is assessed through the number of validated user contributions to the platform. For a certain number of valid fields, a new category will be awarded (Table 1). This is useful to present questions of different difficulty levels to users according to their proficiency (for example, collection date is an easier field than determination). Additionally, the user category is used to attribute confidence to their answers and values submitted by more experienced users have more weight for validation.

**To automatically validate submitted values.** An automatic routine is implemented to evaluate pending submissions for validation. The simple way would be to compare the values for the same field of a specimen submitted by different users; in the case of a match, accept those values. In our system, user levels are based on user contributions (proficiency) and this is utilised to validate data by assigning a confidence value linked to the user status (Table 1). Validation occurs when the sum of points for a value reaches a defined number of points (Table 1). As an example, a value is validated when the sum is equal or higher than 60 points, i.e., a single answer from an administrator is enough to get validation, but it would require six basic users or one expert and one basic user (or any other combination that sums at least 60 points) to submit the same value to be accepted (Table 1).

**To allow submission of any value for any specimen of the collection.** Despite the platform's aim to provide users with sets of specimens organised in missions/projects, the editing properties can be used to edit any specimen. This is useful for data editing by any user consulting the online catalogue. For the edition, a hyperlink in the online catalogue transfers the user to the crowdsourcing platform. For that reason, we populate the platform with all the records in the master database, regardless of the fact that they are integrated or not in a mission/project.

**To fully integrate with the main database.** Data supplied by the crowdsourcing platform are added progressively to the herbarium master database, not waiting for the whole transcribing mission to end, nor even for a specimen to be completed. A script that obtains the list of validated fields from the crowdsourcing platform server (see above for validation methods) through an http request in an incremental way, i.e, it only obtains the records since the last request, obtaining the results in JSON format and importing them into a table. The data integration occurs for each validated value by comparing each one with that present in the SPECIFY master database. When a field is empty (or absent), it is written (or created). Record creation/edition is credited to a specific user in SPECIFY database to allow tracking changes. When a field is already filled in and is equal to the new value, it is considered resolved. If there is a conflict, then it is listed for the administrator to resolve. Conflict resolution is not a live process, since it requires the administrator to check the specimen image for the correct value, being executed later from a control panel where the administrator accepts or rejects values.

## Discussion

The automation suggested in Stage 1 saves considerable human labour and ensures consistency, making the specimens in the online catalogue available with minimal information, which can then be supplemented with the help of the crowdsourcing platform described in Stage 2.

The crowdsourcing platform was intensively tested with ca. 200 students (February to April 2020). Test users were asked to achieve expert level (500 validated answers) in order to evaluate the platform for behaviour and performance. The platform was made publicly available in April 2020 and a few new users have become active since then. Until now, ca. 30,000 fields were validated, being most of the input from the test users. When looking at the validated data, no major problems were detected, despite some inconsistency with our internal patterns (person names or remarks). Nevertheless, those values could be considered safe to integrate the master database and eventually be bulk-corrected in our regular database check-ups. A more detailed quality check analysis will be made in the future, along with other social parameters.

**Figure 3.**
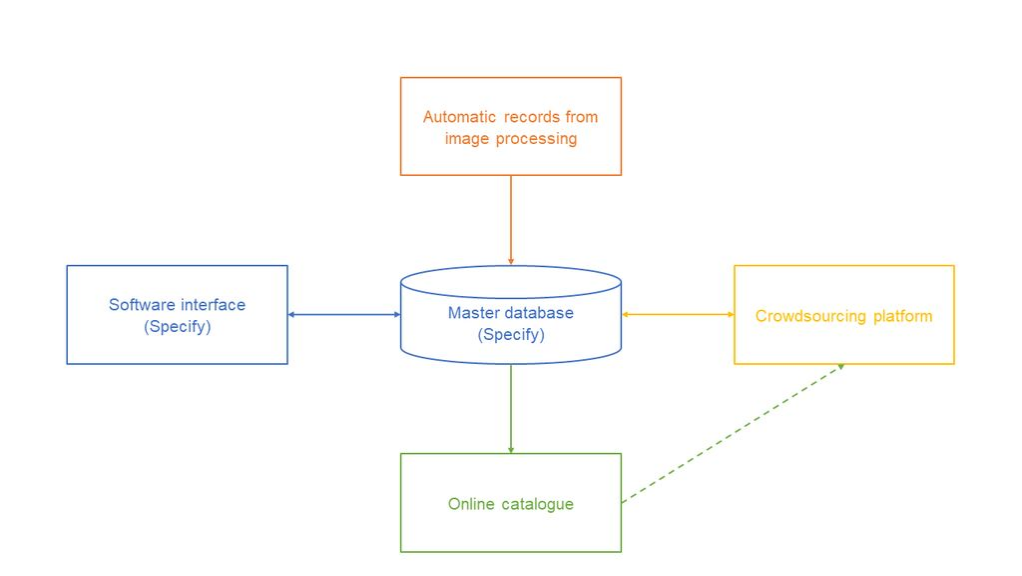
Proposed data flow. Local database (Specify) is managed by its own software interface. Records can also be created automatically from image processing (described in Stage 1) and edited with the crowdsourcing platform. Data is made available in the online catalogue. Any data in the online catalogue can be edited using the crowdsourcing platform.

As online and local systems are integrated, all the information flows across the master database, the online catalogue and the crowdsourcing platform, with very little human intervention (Fig. 3).

The procedures described above will contribute to increasing the rate of digitisation of collections. More than a workflow, this constitutes a paradigm shift, detaching many procedures from the collection staff. Ultimately, most digitisation processes could be run by volunteers, from imaging to validation. This is of utmost relevance in collections with few employees who normally need to be focused on curatorial procedures. Such implementation enables continuous digitisation to occur, even if at low pace, instead of being completely dependent on funded programmes.

## Conclusions

The proposed approach increases the rate of digitisation of specimens. Our automatic databasing procedure reduces the time needed when compared to performing the task manually, i.e. creating the record and then filling up the taxon name.

Validation of crowdsourced data is a sensitive issue because of the risk of inserting incorrect information into the master database. However, the validation method described here, based on the user proficiency, mitigates that risk and we consider that it will give enough confidence to allow the integration of data in the master database.

We emphasise the unique feature of the developed system that enables the edition at any time of any online catalogue data using the crowdsourcing platform.

The integration of the master database, the crowdsourcing platform and the online catalogue results in a novel dynamic environment for data construction. The diverse contributions enrich the final structure.

## Figures and Tables

**Table 1.** Collaborative application: user categories and roles. Validation of submitted data considers user’s proficiency as a criterion of confidence.

**Role**	**Category**	**Description**	**Accepted submissions required**	**Points attributed to each submission**
CONTRIBUTOR	Basic	First time user. Fields displayed are restricted.	0	10
	Beginner	More fields are displayed, but some are restricted.	10	20
	Competent	More fields are displayed, but some are restricted.	50	30
	Advanced	More fields are displayed, but some are restricted.	100	40
	Expert	Can submit all fields.	500	50
ADMINISTRATOR	Administrator	Can perform all tasks above, data management (submission approval)	-	60
ROOT	Root	Can perform all tasks above, administrator management, specimen management.	-	60
